# Supplementary material for: Reference-guided pseudotime inference across species and biological contexts
Source: bioRxiv. 2026 Sep 10:2026.09.04.749461. Preprint. [Version 1] doi: 10.64898/2026.09.04.749461 (PMC13622487; doi:10.64898/2026.09.04.749461)
Supplement: Supplementary file 1 [file NIHPP2026.09.04.749461V1-supplement-1.pdf]

bioRxiv preprint doi: https://doi.org/10.64898/2026.09.04.749461; this version posted September 10, 2026. The copyright holder for this preprint (which was not certified by peer review) is the author/funder, who has granted bioRxiv a license to display the preprint in perpetuity. It is made available under a CC-BY 4.0 International license.

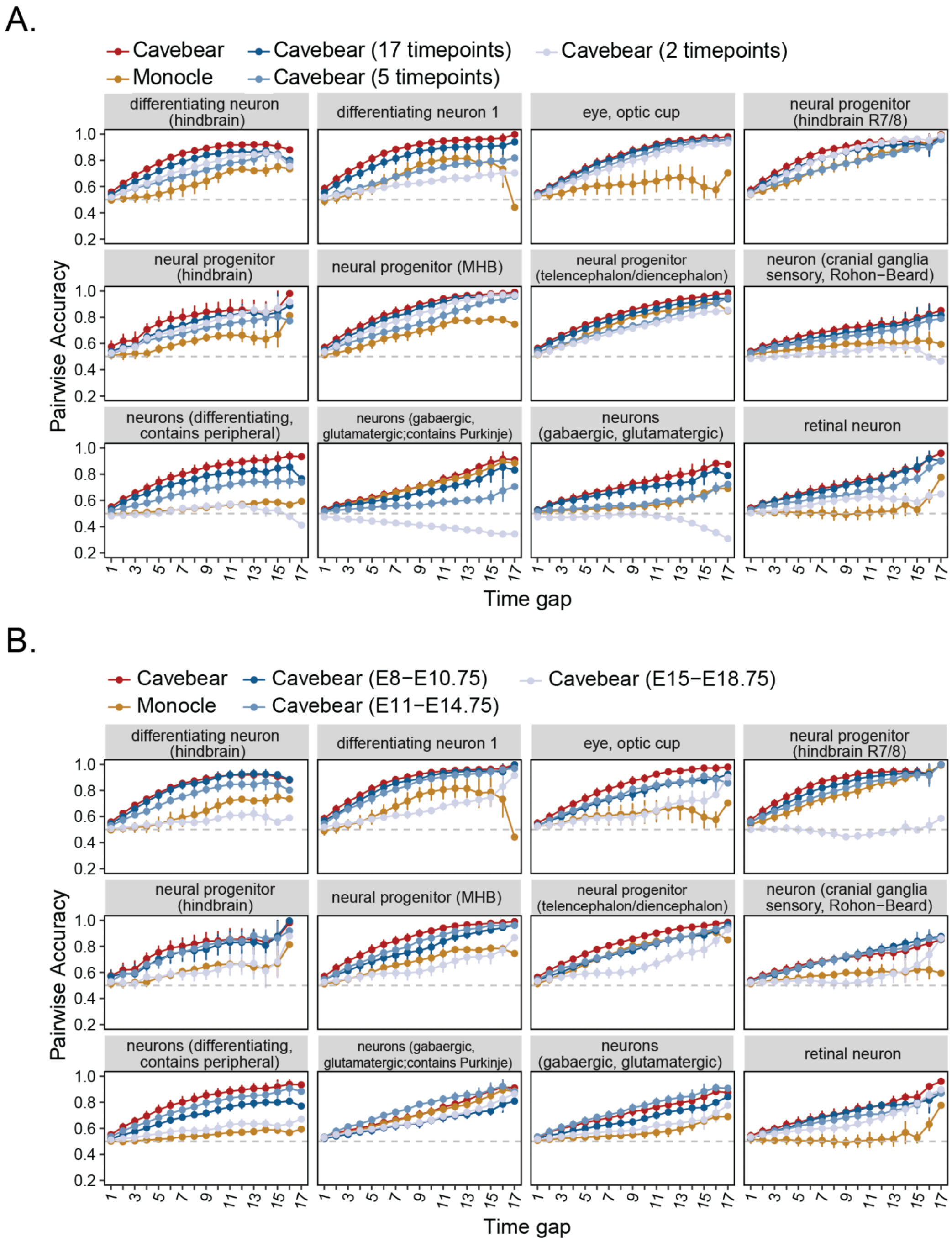

Figure S1: Cavebear's performance is affected by timepoint density and coverage.
(A) Accuracy of Cavebear predictions in recapitulating temporal order between pairs of cells. Each color represents a prediction method. Cavebear represents the full model trained on all 67 timepoints in reference, and methods colored in blue show subsetted Cavebear models covering a reduced number of timepoints. Monocle is shown as a comparison baseline. **(B)** Similar to A, the Cavebear model is trained on reference timepoints within three time periods, colored in different shades of blue, to capture how changing reference periods affect results.

bioRxiv preprint doi: https://doi.org/10.64898/2026.09.04.749461; this version posted September 10, 2026. The copyright holder for this preprint (which was not certified by peer review) is the author/funder, who has granted bioRxiv a license to display the preprint in perpetuity. It is made available under a CC-BY 4.0 International license.

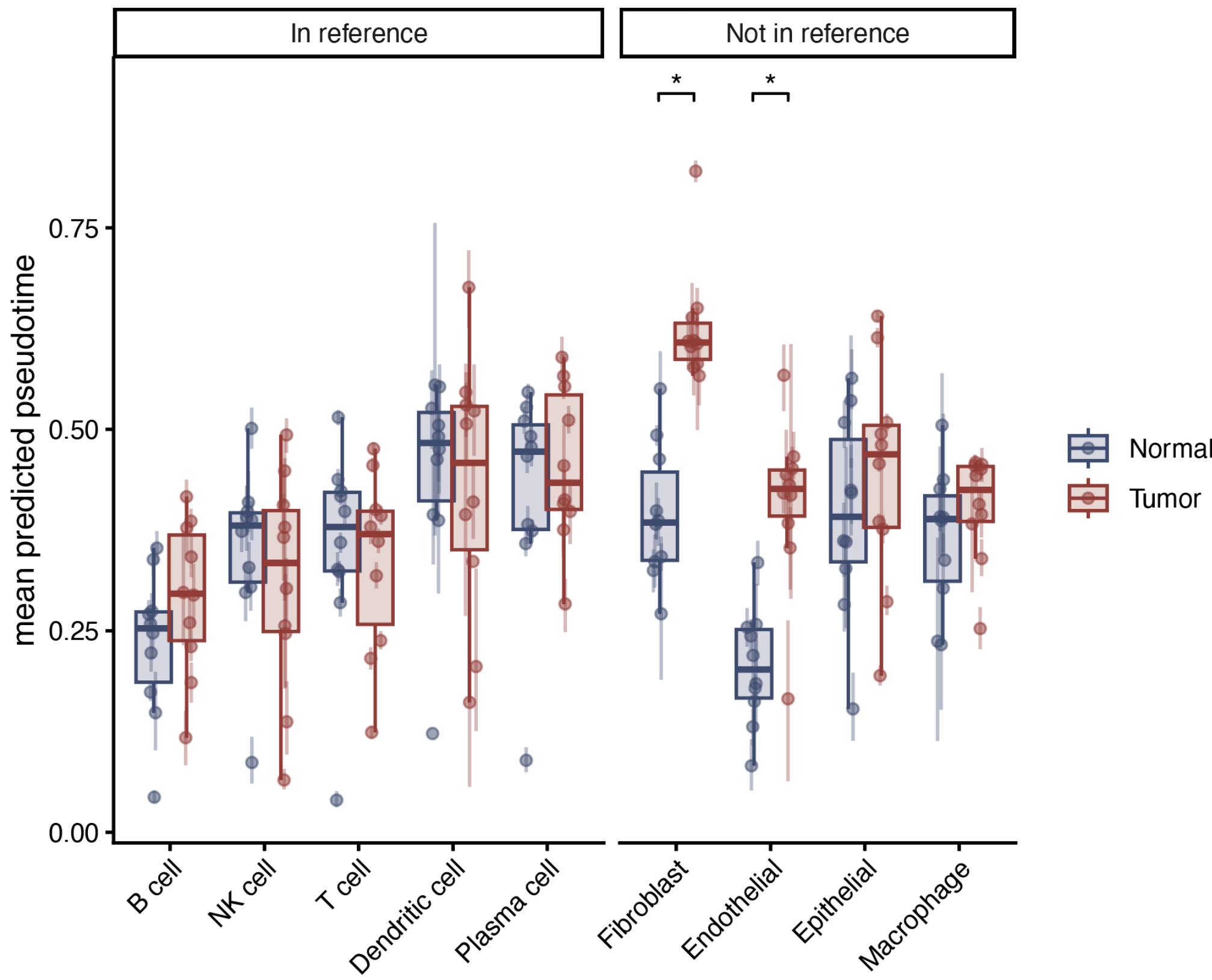

Supplementary Figure S2. Cancer progression signals in a human colorectal target dataset can also be detected when using a CML mouse reference. (indicates adjusted p-value at different levels of significance: ** < 0.01, * <= 0.05)
